# Pre-Fibrillar α-Synuclein Mutants Cause Parkinson's Disease-Like Non-Motor Symptoms in *Drosophila*

**DOI:** 10.1371/journal.pone.0024701

**Published:** 2011-09-08

**Authors:** Madhu Babu Gajula Balija, Christian Griesinger, Alf Herzig, Markus Zweckstetter, Herbert Jäckle

**Affiliations:** 1 Abteilung Molekulare Entwicklungsbiologie, Max-Planck-Institut für biophysikalische Chemie, Göttingen, Germany; 2 Abteilung für NMR-basierte Strukturbiologie, Max-Planck-Institut für biophysikalische Chemie, Göttingen, Germany; University of Texas M. D. Anderson Cancer Center, United States of America

## Abstract

Parkinson's disease (PD) is linked to the formation of insoluble fibrillar aggregates of the presynaptic protein α-Synuclein (αS) in neurons. The appearance of such aggregates coincides with severe motor deficits in human patients. These deficits are often preceded by non-motor symptoms such as sleep-related problems in the patients. PD-like motor deficits can be recapitulated in model organisms such as *Drosophila melanogaster* when αS is pan-neurally expressed. Interestingly, both these deficits are more severe when αS mutants with reduced aggregation properties are expressed in flies. This indicates that that αS aggregation is not the primary cause of the PD-like motor symptoms. Here we describe a model for PD in *Drosophila* which utilizes the targeted expression of αS mutants in a subset of dopadecarboxylase expressing serotonergic and dopaminergic (DA) neurons. Our results show that targeted expression of pre-fibrillar αS mutants not only recapitulates PD-like motor symptoms but also the preceding non-motor symptoms such as an abnormal sleep-like behavior, altered locomotor activity and abnormal circadian periodicity. Further, the results suggest that the observed non-motor symptoms in flies are caused by an early impairment of neuronal functions rather than by the loss of neurons due to cell death.

## Introduction

Parkinson's disease (PD) correlates with the formation of insoluble fibrillar aggregates in the central nervous system that contain α-Synuclein (αS) [1], [2]. Importantly, misfolding of αS and aggregation of the protein can be aggravated by point mutations in the αS gene. Mutations that alter the αS fibrillation characteristics, such as the A53T mutation [3] were found to cause an autosomal dominant form of PD [3]–[5]. In addition to A53T, a number of αS mutants with impaired ß-structure have been generated and shown to aggregate much later than wild type αS [6]. Pan-neuronal expression of these mutant αS proteins, which aggregate less efficiently than wild type αS, causes PD-like motor symptoms more efficiently than wild type αS when expressed in neurons of model organisms such as *Drosophila melanogaster* (reviewed in [7]) and *Caenorhabditis elegans* [6]. The most effective pre-fibrillar αS mutant tested so far consists of three alanine replacements by prolines (at positions A30P, A56P and A76P; “TP-αS”). TP-αS is strongly impaired in amyloid fibril formation and fails to aggregate [6]. This observation suggests that soluble pre-fibrillar αS oligomers are in fact responsible for causing motor symptoms observed in PD and other neurodegenerative diseases, in agreement with earlier proposals [8], [9].

Interestingly, motor-impairment in PD patients is known to be often preceded by non-motor symptoms such as sleep problems. Therefore, we asked whether pre-fibrillar αS mutations can also induce PD-like non-motor symptoms in *Drosophila*. We expressed αS mutant protein in a subset of neurons, that includes the serotonergic and dopaminergic (DA) neurons of *Drosophila* and flies were examined for alterations in their sleep behavior and circadian activity. Here we report that the expression of pre-fibrillar αS mutant proteins does indeed cause non-motor symptoms in flies; these include an altered sleep-like rest behavior [10], an extended circadian rhythm, as well as an abnormal locomotion. Importantly, these symptoms were all observed prior to cell death, strongly suggesting that it is the early impairment of the DA system, by the pre-fibrillar αS proteins, that is responsible for the observed non-motor symptoms. Our results present a powerful genetic model for PD, allowing the dissection of the underlying mechanisms by which non-aggregating αS impairs the DA system and causes non-motor symptoms.

## Results

Recent results have shown that pan-neuronal expression of pre-fibrillar oligomers of mutant αS causes PD-like motor symptoms in *Drosophila* [6]. Importantly, these mutant αS proteins show reduced fibrillization propensity, but form increased amounts of soluble oligomers in comparison to aggregating wild type αS [6]. One of the αS mutants, A53T-αS, is linked to familial PD [3]. It forms pre-fibrillar αS oligomers that aggregate later than wild type αS [6]. A second mutant, the triple alanine to proline mutation TP-αS forms pre-fibrillar oligomers but fails to aggregate. Upon pan-neuronal expression, the strength of the motor symptoms and cell toxicity correlate with the aggregation properties of the protein (wild type αS<A53T-αS<TP-αS; details in [6]).

In order to explore whether expression of the TP-αS and A53T-αS mutant proteins causes PD-like non-motor symptoms in flies, we employed the bipartite UAS/Gal4 expression system [11] using the *Ddc-Gal4* [12] and *TH-Gal4* [13] drivers. They allow UAS-dependent αS mutant transgene expression in both serotonergic and dopaminergic (DA) neurons [12] and in DA neurons [13], respectively. Positional effects can complicate analysis of transgenes, particular when transgenes have inserted randomly into different positions and are subject to varying positional effects. Therefore, to precisely insert the different transgenes into the same pre-determined chromosomal locations, we used the φC31-based site-specific recombination system [14]. As controls for the possible effects αS mutant, we expressed wild type αS (WT-αS) and bacterial *lacZ* under otherwise identical conditions.

### Expression of αS mutants affects the sleep-like rest behavior of flies

The *Ddc-Gal4*-dependent expression of αS or mutant αS proteins in flies and resulting PD-like motor symptoms have been described (see [6], [15]). However, non-motor symptoms, such as the abnormal sleep behavior seen in PD patients [16], [17], have not been addressed in a simple animal PD model organism. Therefore, we asked whether the sleep-like rest of flies [10], to which we refer to as “sleep”, is affected in flies expressing wild type αS, A53T-αS or TP-αS.

**Figure 1:**
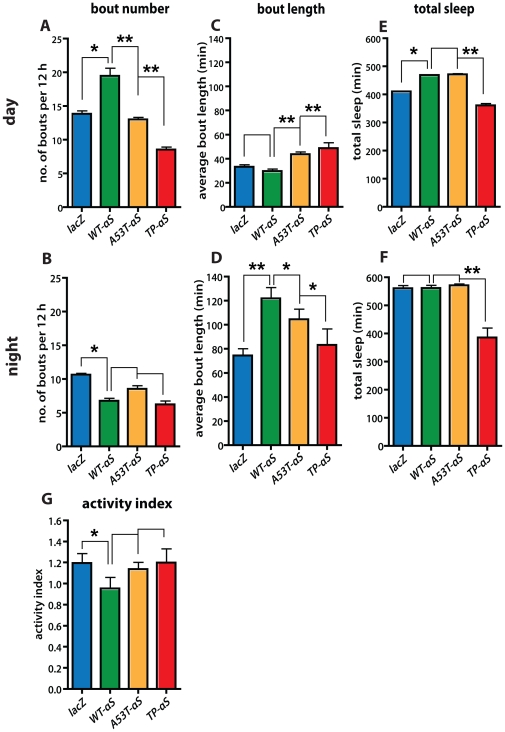
Sleep behavior of flies expressing *lacZ*, wild type αS and αS mutants from *Ddc-Gal4*-driven UAS transgenes. Expression of pre-fibrillar αS mutants (A53T-αS, TP-αS; see text) in young flies (3 days after hatching) affect the average number of sleep episodes (“bout number”; **A**, **B**), the average length of sleep episodes (“bout length”; **C**, **D**), and the total length of sleep (“total sleep”; **E**, **F**) more severely than αS expression both during the light (“day”; **A**, **C**) and dark (“night”; **B**, **D**) periods. The activity level during the wake periods (“activity index”; **G**) were not significantly affected. Bars represent mean values of at least three independent experiments ± the standard error of the mean from 32 animals that were individually recorded in each experiment. Differences among means were determined by one-way ANOVA followed by the Newman-Keuls Multiple Comparison *post hoc* test using wild type αS (Wt-αS) as control for the mutants; no asterisk: *P*≥0.05; one asterisk: *P*<0.01; two asterisks: *P*<0.001. *lacZ* and Wt-αS refer to *lacZ* and wild type αS control expression; A53T-αS and TP-αS to the αS mutants; for details see text.

Sleep in flies includes canonical features of mammalian sleep [10], [18]–[20]. We tested whether the number of sleep episodes (“bout number”), the length of sleep episodes (“bout length”) and the total length of sleep (“total sleep”) [21], [22] is affected in flies expressing mutant forms of αS. Flies were raised and kept under a 12 hrs light/12 hrs day cycle (L∶D = 12 hrs∶12 hrs) (Fig. 1A–F). Control flies expressing *lacZ* or wild type αS and flies expressing A53T-αS or TP-αS were examined three days after hatching (“young flies”).

When compared to *lacZ* expression, the expression of wild type αS led to a higher number of sleep episodes during the light phase (“bout number”; Fig. 1A), but the average length of each sleep episode (“bout length”) was not significantly affected (*P*≥0.05; Fig. 1C). During the dark phases, wild type α-S expression led to a reduction of bout number (Fig. 1B) but an increase in bout length (Fig. 1D) when compared to control flies (*lacZ* expression). Expression of A53T-αS and TP-αS led to a mild and strong increase in both bout number and bout length, respectively, when compared to *lacZ* expression during the light and dark phases (Figure 1A–D). Taken together, the results show that while expression of wild type αS interrupts the sleep of flies during the day more frequently than occurs in *lacZ* expressing control flies. In contrast, the expression of the two α-S mutants had an opposite effect (TP-αS>A53T-αS), i.e. the number of sleep episodes was reduced during the day (Fig. 1A), but the average length of the sleep episodes was extended (Fig. 1C). During the night, both wild type αS and the αS mutants reduced the number of sleep episodes experienced by the flies (Fig. 1B), and the length of the sleep episodes (Fig. 1D) was extended (αS>A53T>TP-αS). While the expression of wild type αS and A53T-αS had no significant effect on the total sleep of the flies, the expression of the TP-αS mutant led to a significant reduction in the time the flies spent asleep during a 24 h period (*P<0.001*; Figure 1E, F). However, the level of activity in awake flies expressing wild type or mutant αS was not significantly affected (P≥0.05) (Fig. 1G; “activity index”; see Materials and Methods).

The results indicate that the sleep behavior of flies is altered in a protein specific manner in response to αS, A53T and TP-αS expression. In human PD patients, difficulty in sleep maintenance (sleep fragmentation) is the earliest and most frequent sleep disorder in such patients reported [16], [17]. Other common sleep-associated complains include excessive daytime sleepiness. However, a systematic exploration of sleep problems in human patients has never been undertaken, and the etiology of the problems remains unknown. Furthermore, it is also unclear whether some of the sleep problems of PD patients are actually related to the disease process itself or rather to side-effects from therapeutic strategies employed [16], [17]. Thus, it is not surprising that the expression of αS and the αS mutants results in a variable pattern of sleep abnormalities when compared to *lacZ* control gene expression. However, it is important to note that the effects in each experimental series can be crudely ordered according to the biophysical properties of the proteins, i.e. their propensity to form pre-fibrillar αS oligomers (A53T<TP-αS) instead of aggregates (αS) (see Fig. 1A, C, D).

In summary, these findings establish that αS expression affects the sleep behavior of young flies prior to the stage when motor symptoms and neurotoxicity of the mutants can be detected [6], [15] (and our own observations). Thus, abnormal sleep in young flies is likely to be caused by dysfunctional neurons system, resulting from αS as well as αS mutant protein expression, and not only by their degeneration which is observed concomitant with motor syndromes in the older flies [6], [15].

### Expression of the αS mutants affects circadian locomotor activity

**Figure 2:**
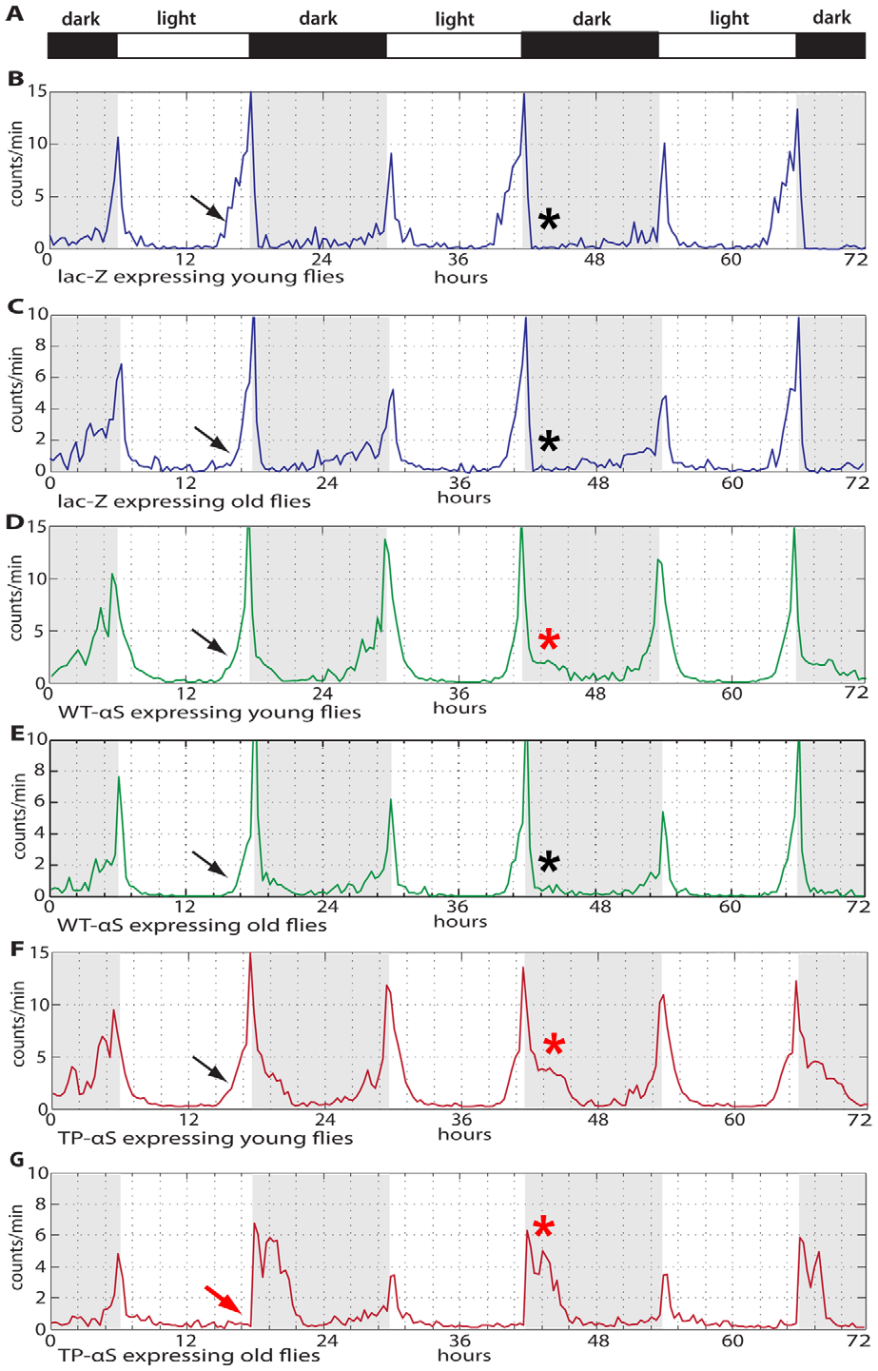
Locomotor activity and anticipation of the dark-light transition of flies expressing *lacZ*, wild type αS and the oligomer-forming TP-αS mutant. (**A**) Dark-light (D∶L = 12 hrs∶12 hrs) transition bar. (**B–G**) Locomotor activity profile of young flies (3 days after hatching; **B**, **D**, **F**) and old flies (30 days after hatching; **C**, **E**, **G**); expressing *lacZ* (blue line; **B**, **C**), WT-αS (green line; **D**, **E**) and TP-αS (red line; **F**, **G**). Black arrows point to the beginning of locomotor activity prior to the onset of light (anticipatory behavior of the flies). Note that old flies expressing TP-αS fail to anticipate the onset of the light period (red arrow in **F**). Red asterisks show the phasing out of the maximum locomotor activities after the light-dark switch. For details see text.

The dopaminergic neuronal system of *Drosophila* is predicted to play a role in circadian entrainment and in translating the circadian molecular oscillations in clock cells into locomotor activity of the organism [23], [24]. We next asked whether the circadian locomotor activity is affected by *Ddc-Gal4* mediated pre-fibrillar αS expression and whether the flies can anticipate the dark-light (D∶L) transition after a D∶L cycle entrainment (12 hrs∶12 hrs; Fig. 2A) as reported for wild type flies [25]. This anticipation behavior of the flies is reflected in a slow increase in locomotor activity prior to the D∶L transitions (arrows in Fig. 2B–G).

*Ddc-Gal4-*driven expression of *lacZ* in young and old flies had no detectable effect on the circadian locomotor activity profiles of the flies (Fig. 2B–C). *Ddc-Gal4-*driven expression of wild type αS (Fig. 2D,E), and in particular TP-αS expression (Fig. 2F,G), altered the locomotor activity profiles. The anticipation behavior was maintained in *lacZ* expressing young (1–3 days after eclosion) and old (30 days after eclosion) control flies (Fig. 2B, C) as well as in young and old flies expressing wild type αS (Fig. 2D, E). It was also observed with young flies expressing the TP-αS mutant protein, but old flies lost this behavioral characteristic in response to TP-αS expression.

Furthermore, there is normally a maximum activity peak before the L∶D transition followed by a sharp decrease (asterisk in Fig. 2 B–G), indicating that “sudden dark” represents a signal for flies to abruptly stop their locomotion. In young flies, αS expression causes a phasing out of the locomotion activities (asterisk in Fig. 2 D) which is even more pronounced in flies expressing TP-αS (circle in Fig. 2F). This effect is further enhanced in old flies that express the mutant protein (asterisk in Fig. 2 G), a phenomenon that is not observed with wild type αS expressing flies. One possible explanation for this unexpected recovery in αS expressing but not in TP-αS expressing flies is that soluble, pre-fibrillar αS impairs neuron function, whereas αS aggregates which form in αS, but not in TP-αS expressing cells [6], have no such effect. In fact, 30 days after fly eclosion, the majority of αS expressing neurons contain such aggregates, but no massive cell death can be detected [15]. In contrast, the high toxicity of the TP-αS mutant causes massive amounts of cell death [6]. This interpretation would imply that the presence of αS oligomers impairs neuronal function even in young flies, with severe age-related phenotypes perhaps reflecting the high levels of accompanying cell death. In contrast, alive cells containing wildtype αS aggregates retain a higher level of functionality for longer periods due to lower neurotoxicity of αS.

**Figure 3:**
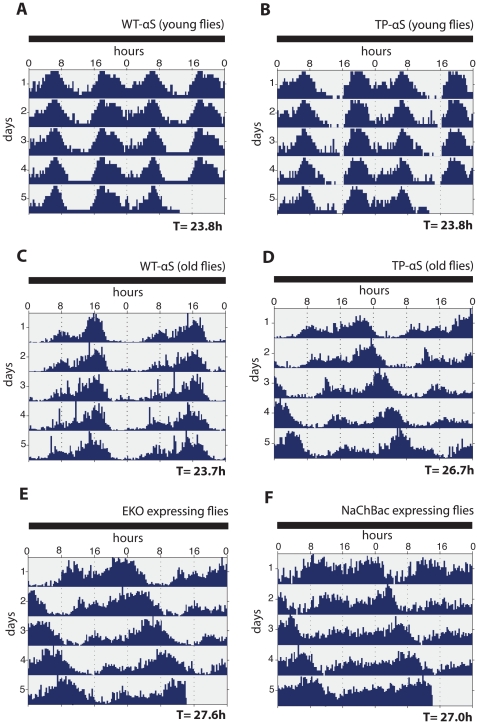
Age-dependent circadian defects in response to wild type αS, TP-αS, EKO/Kir2.1 and NaChBac expression in DA neurons. (**A**, **B**) Double-plotted actograms of young flies (3 days after hatching) expressing wild type αS (WT-αS) (A) and TP-αS (B) under the control of the DA neuron specific *TH-Gal4* driver. T refers to circadian periodicity which is 23.8 hrs in both cases. (**C**, **D**) Double-plotted actograms of old flies (30 days after hatching) expressing WT-αS and TP-αS. Note the different circadian periodicity in response to WT-αS (T = 23.7 hrs) and TP-αS expression (T = 26.7 hrs). (**E**, **F**) Double-plotted actogram of old flies expressing EKO/Kir2.1 (E) or NaChBac (F) under the control of the DA neuron specific *TH-Gal4* driver. Note the similar extension of the circadian periodicities (T = 27.6 hrs and T = 27.0 hrs, respectively) as observed after TP-αS expression. All experiments (n = 32–58 flies) were carried out under constant dark conditions after the animals were kept in a dark-light cycle of 12∶12 hrs. T was calculated by the Chi-squared periodogram analysis (see Materials and Methods).

In order to assess a possible effect of TP-αS expression on the circadian rhythms of the flies, we monitored the circadian locomotor activities under constant dark conditions (DD) with young and old flies, respectively. Flies were raised and kept for three days in a L∶D cycle of 12 hrs light and12 hrs dark. Flies were then transferred to continuous dark conditions (young flies) or kept an additional 27 days in the L∶D before being transferred to continuous dark (old flies). Actograms for both sets of flies were obtained over a a period of ten days (Fig. 3A–D). Fig. 3A and B show that the normal circadian activity of young flies is not altered in response to either αS or TP-αS expression, i.e. they maintain their normal periodicity (T = 23.8 hrs) over a period of 10 days. Old flies expressing αS also maintain a normal periodicity (T = 23.7 hrs), whereas old flies expressing TP-αS shift their periodicity (T = 26.7 hrs) similar to flies which lack key components of the circadian clock control such as *period* [26]. These findings establish that TP-αS expression in neurons interferes with the circadian rhythm of aging flies.

### DA neuron inactivation causes a TP-αS-like locomotor activity phenotype

The DA system plays an as yet undefined role in regulating the circadian locomotor activity of flies [23], [24]. We sought to test whether the circadian defects observed after TP-αS expression relate to the DA system, and if so, whether they are due to neuronal dysfunctions or neural degeneration. To accomplish this, we reduced the activity in DA neurons either by expressing a mutant Shaker potassium channel (EKO/Kir 2.1) or a bacterial sodium channel (NaChBac) from UAS-cDNA transgenes driven by the DA-specific *TH-Gal4* driver [12]. Both ion channels attenuate synaptic transmission from DA neurons by interfering with their membrane excitability, but the DA neurons remain viable instead of undergoing cell death [27], [28] as has been observed with DA neurons after expressing wild type αS protein or αS mutants [6], [15] (and our own observations). Figs. 3E, F show that the expression of EKO/Kir 2.1 and NaChBac in DA neurons caused a phase shift in circadian locomotor activity and gradually extended the circadian periodicity, as also observed in response to TP-αS expression (Fig. 3E, F). These observations suggest that the circadian effects observed with EKO/Kir 2.1 and NaChBac expressing flies are due to dysfunctional DA neurons rather than the result of their elimination by cell death.

## Discussion

Our results provide evidence that the expression of pre-fibrillar αS oligomers causes non-motor symptoms in *Drosophila*. We made use of an experimentally designed αS mutant protein, the TP-αS protein, which fails to aggregate both *in vitro* and *in vivo* [6]. This protein contains features similar to the A53T-αS mutant protein that is linked to familial PD [3], but is more neurotoxic and causes more severe PD-like motor symptoms when expressed in model organisms [6]. TP-αS expression also produces more severe non-motor symptoms in flies. It interferes with the sleep-like rest behavior of flies (Fig. 1), their anticipation of the dark/light transition (Fig. 2) and with the circadian periodicity (Fig. 3). These symptoms precede the onset of motor symptoms in flies and can be related to the impaired ß-structure of the TP-αS protein which prevents protein aggregation (see also [6]).

Sleep abnormalities in PD patients [29], [30] can be diagnosed years before motor syndromes appear [20], [31]–[33]. Expression of mutant αS in DA neurons also causes sleep abnormalities in young flies, both prior to the appearance motor deficits and before eventual neuronal cell death [6], [15]. This finding suggests that the abnormal rest behavior of flies, a sleep-like state with characteristics of mammalian sleep [10], [18]–[20], is actually caused by the dysfunction of neurons rather than their degeneration; although degeneration does gradually increase in aging flies [6], [15]. Further, it provides support for the proposal that PD symptoms are already initiated by pre-fibrillar oligomer formation of αS prior to its eventual aggregation. The observation that flies expressing TP-αS lose the capability to anticipate the onset of light (see Fig. 2G) confirms also a link between abnormal sleep behavior and memory deficits that have been described earlier [34]. It also suggests that DA neurons in flies, like the DA neurons in mammals, have a role in the modulation of the sleep-wake transition [35], [36].

In addition to sleep, TP-αS expression in DA neurons also affects circadian periodicity and locomotor activity in an age-dependent manner. Unfortunately, these non-motor effects are only observed in aged flies (see Fig. 3) at a stage when neuron degeneration is already apparent [6], [15]. Thus, it remaines unclear whether these effects are due to dysfunctional DA neurons, or their actual cell death. However, we anticipate that these disorders result initially from synapse dysfunction; attenuating synaptic transmission, either by expression of EKO/Kir 2.1 or NaChBac [27], [28] produces symptoms similar to those observed after TP-αS expression. Our results also support the recently proposed role of the DA system in regulating circadian locomotor activity [23], [24]. However, our results cannot exclude the possibility that the neurotoxic activities of αS [6], [15] and the αS mutants [6] may actually underlie the increasingly severe phenotypes seen in aging flies.

TP-αS expression in DA neurons interferes with the circadian rhythm of aging flies, thus showing an intriguing similarity to symptoms reported for patients suffering from severe PD (e.g. [37]). In addition, TP-αS expression, and to a lesser extent expression of the human A53T-αS mutation, affects the sleep behavior of flies. These similarities suggest that αS-dependent non-motor symptoms of PD are recapitulated in flies. Moreover, the circadian clock and DA neuronal signaling mechanisms that regulate arousal and sleep/wake cycles are conserved between mammals and *Drosophila* [38]. Thus, *Drosophila* can be used as a valid model for PD. Importantly, it will be possible to answer questions that cannot be addressed in humans; the fact that different αS mutants can be expressed from the same chromosomal site may help to examine the mechanisms and even quantitative aspects of symptom-causing αS mutants in order to not only elucidate the cause of PD-like motor symptoms but also how they relate to the preceding non-motor symptoms.

## Materials and Methods

### Generation of αS mutations, transgenic flies and fly stock keeping

αS, *lacZ* and αS mutant transgenes (A53T-αS, TP-αS) were generated as described [6] using the *Gal4*-responsive pUAST expression vector containing the attachment site B (*attB*) [14]. Transgene DNA was injected into fly embryos which were double homozygous for both an attachment site P site (*attP*) and the germ-line-specific φC31 integrase. Site-specific integration of transgenes was verified by PCR using the set of primer pairs described [6].

For targeted transgene expression, we used the UAS/Gal4 system [11] with *Ddc-Gal4* [12] and *TH-Gal4* driver lines [13] as previously described [39]. Flies were routinely kept at 25°C on standard fly food [40] using a 12 hs dark/12 hs light (D∶L) cycle unless otherwise stated in the Results.

### Sleep and circadian behavior assays

Flies were housed under a D∶L (12 hrs∶12 hrs) cycle at 25°C with equal population densities. Locomotor activity was recorded from single males using the *Drosophila* Activity Monitoring (DAM) system (Trikinetics, Waltham, USA) as described [21], [22]. Briefly, the DAM contains 32 channels, each connected to a single small glass tube, in which the activity of individual flies can be monitored, as they “break” an infrared beam that bisects the tube by moving back and forth along the container. Movements were recorded in 1 min bins. Sleep is defined as a bout of 5 or more minutes of inactivity [18]. The average length of a sleeping bout was calculated as the total amount of sleep (in min.) divided by the number of sleep periods. Thectivity index refers to the total number of recorded movements in the DAM system divided by the total time (in min.) that flies were awake.

To assess circadian locomotor activity, single males were individually recorded (32 per experiment) using the DAM system. Three independent experiments were performed for each genotype. Animals were kept in D∶L conditions (12 hrs∶12 hrs), before they were shifted to a dark only environment. Data were analyzed with Clock Lab algorithms to extract the circadian behavior (Actimetrics, Wilmette, USA). Double-plotted actograms, Chi-squared or Lomb-Scragle periodograms were plotted using MATLAB 7.4 (R 2007a) software using the Clock Lab programs (Actimetrics, Wilmette, USA).
